# Editorial: One health approach to infection prevention and antimicrobial resistance

**DOI:** 10.3389/fcimb.2026.1935103

**Published:** 2026-08-05

**Authors:** Roula M. Abdel-Massih, Ramy K. Aziz, Ziad Daoud

**Affiliations:** 1 College of Medicine, Central Michigan University, Mount Pleasant, MI, United States; 2 BioRAM Consulting, Public Health and Biorisk Management Consultancy, Midland, MI, United States; 3 Department of Microbiology and Immunology, Faculty of Pharmacy, Cairo University, Cairo, Egypt; 4 Bioscience Research Laboratories, MARC for Medical Services and Scientific Research, Giza, Egypt; 5 Department of Medical Laboratory, MyMichigan Health System, Midland, MI, United States

**Keywords:** antimicrobial resistance (AMR), antimicrobial stewardship, clinical microbiology, genomic surveillance, infection prevention, One Health

## 1 Introduction: one microbiology for One Health

Since its inception, less than two centuries ago, microbiology has radically transformed how infectious diseases are understood and managed. The germ theory, and the subsequent identification of causative disease agents and their routes of transmission laid the foundation for public health measures of disease prevention and control. Microbiology has conveniently been subdivided into medical and environmental branches. However, two major conceptual shifts have brought those branches together: one is biocentric, the advent of microbiome research, which views human hosts as just another ecosystem for microbes, making the separation between medical microbiology and microbial ecology artificial: the health of all ecosystems, whether inanimate or biotic, is substantially affected by microbial forms. The other conceptual shift is anthropocentric and highly relevant to public health: the ‘One Health’ framework, featured in this Research Topic, which sees a tight connection between the health of humans, animals, and the environment.

Indeed, the health of humans, animals, and the environment are so interconnected that they cannot be studied separately. This cannot be more applicable than to the antimicrobial resistance (AMR) crisis. The overuse of antimicrobials in medical, veterinary, and agricultural applications, together with poor infection control practices and environmental pollution, are all factors accentuating AMR. This *R*esearch *T*opic particularly focuses on integrated surveillance systems, novel prevention measures, and antimicrobial stewardship programs that engage multiple sectors. It includes 14 articles by 120 authors, covering three major axes: an epidemiological axis, a diagnosis/clinical axis, and a stewardship/awareness axis.

## 2 One health epidemiology: tracing AMR transmission with molecular and genomic tools

Developing effective diagnostics, preventive strategies, and interventions against AMR requires a comprehensive understanding of the emergence, persistence, and transmission of resistance factors across human-animal-environmental interactions highlighting the importance of a One Health approach. In one set of papers in this special topic, researchers focus on integrating molecular or genomic surveillance as well as inter-sectoral collaboration to monitor and control the spread of AMR, notably at the human-animal interface. In a post-COVID world, it is unthinkable to envisage epidemiology without genomic surveillance.

In a One Health framework, genomic or genetic surveillance allows tracking the spread of resistant clones between humans and animals (e.g., humans *vs.* their companion animals in the USA (Dietrich et al.) and animal handlers *vs.* dairy herds and milk products in India (Vijay et al.)). A closer focus on the nature of AMR transmission, i.e., whether it is vertically (mutation-driven) or horizontally transferred (via plasmids, phages, or integrons), allows better diagnostic and interventional measures. This has been conducted meticulously by Luo et al. through sequencing the genomes of 140 carbapenem-resistant *Klebsiella pneumoniae* isolates and tracing their spread across wards in a tertiary care hospital in China, with a focus on the expansion of plasmid-associated resistance.

Although whole genome sequencing is attractive, it is not the only way to trace AMR, and its cost and elaborate analysis may be sometimes prohibitive. Alternative molecular approaches, such as quantitative polymerase chain reaction (qPCR) or droplet digital PCR (ddPCR) are useful for absolute quantification of bacterial cells, and can be used in some cases to define the viable-but-non-culturable (VBNC) state of some bacterial strains (e.g., alcohol-producing *Klebsiella pneumoniae* (Zhao et al.), as detailed below).

## 3 One Health for diagnosis and clinical decision support

Clinical microbiology is changing rapidly. It is no longer defined solely by identifying pathogens and reporting susceptibilities. Nowadays, microbiology laboratories play a more central role in patient care, influencing antimicrobial stewardship, infection prevention, quality improvement, and public health.

A clear example of this evolution is the article by Wang et al.. By combining MALDI-ToF-MS identification, 24/7 blood culture processing, and automated preliminary susceptibility reporting, the investigators significantly shortened the time to Gram stain reporting, organism identification, and susceptibility results. These improvements are not simply operational: in bloodstream infections, faster microbiology means faster optimization of antimicrobial chemotherapy and a more active role for the laboratory in stewardship and precision care.

The study optimizing the bedside precleaning protocols for colonoscopes (Li et al.) shows how microbiology-informed process improvement can strengthen infection prevention. By systematically evaluating precleaning variables and demonstrating that ATP bioluminescence correlates closely with microbial culture results during the cleaning phase, the investigators provide a practical framework for improving processing quality. Similarly, the study of Wang et al. shows how strengthening environmental hygiene practices can contribute to AMR mitigation. The free provision of disinfectant wipes combined with bundle management reduced detection rates of multidrug-resistant organisms (MDROs), hospital-acquired infections, and mortality.

While these two studies emphasize speed and process control, the study by Li et al. highlights an important frontier: prediction. Using only three readily available clinical variables, the investigators developed a practical scoring system to identify ICU patients at increased risk for carbapenem-resistant *Acinetobacter baumannii*. In an era of rising resistance and pressure to use broad spectrum therapy more judiciously, such tools may help clinicians make more informed empiric decisions while reducing unnecessary carbapenem exposure. This is where microbiology and stewardship converge not only in confirming and reporting resistance, but in helping anticipate it.

The field is also expanding beyond the hospital setting. Dietrich et al. underscore the importance of a One Health approach to antimicrobial resistance surveillance. By linking genomic and epidemiologic data across human and companion animal isolates, the study demonstrates both the promise and the complexity of tracking resistance across interconnected reservoirs, relaying a clear message: understanding the spread of resistant organisms requires stronger collaboration among clinical laboratories, public health, and veterinary medicine. Similarly, Zhang et al. highlighted the importance of continuous molecular surveillance by documenting shifts in rhinovirus genotypes following the COVID-19 pandemic.

Finally, Zhao et al. address a long-standing blind spot in microbiology: VBNC organisms. By combining propidium monoazide with ddPCR, the investigators developed a method to directly quantify viable *Klebsiella pneumoniae* in the VBNC state. The significance of this work goes beyond technical refinement. VBNC organisms may contribute to persistence, recurrence, and ongoing transmission despite negative cultures, and methods that better detect these hidden populations may reshape our understanding of chronic infections and treatment response.

Taken together, these studies capture the direction in which clinical microbiology is moving. The laboratory is becoming faster in delivering actionable results, more rigorous in improving care processes, more predictive in supporting antimicrobial decisions, more connected to One Health surveillance, and more sophisticated in overcoming the limitations of conventional culture.

The future of clinical microbiology cannot and should not be defined by any single technology, but by the integration of rapid diagnostics, process optimization, predictive tools, genomic surveillance, and innovative molecular methods into everyday practice. These studies offer a compelling example of that future and, more importantly, a reminder of why it matters; better microbiology does not just improve laboratory performance but improves patient care.

## 4 Stewardship, education, communication, and future interventions

The fight against AMR requires more than the discovery of new antimicrobial agents. AMR mitigation requires a One Health approach that integrates innovative prevention interventions across humans, animals, and the environment. It also depends on effective antimicrobial stewardship, professional and community-based educational programs, and evidence-based communication tailored to diverse audiences.

One of the main challenges in AMR control is translating knowledge into sustainable behavioral change and evidence-based practice. Despite increasing recognition of One Health principles, there is a disconnect between theoretical understanding and practical implementation across human, animal, and environmental health sectors. The study evaluating clinicians’ knowledge, attitudes, and practices (KAP) regarding pediatric group A *Streptococcus* pharyngitis management in Shenzhen, China (Zhuang et al.) demonstrated generally positive attitudes, but also identified important discrepancies between knowledge and clinical practice. Although clinicians were generally familiar with evidence-based management, their clinical behavior was sometimes constrained by individual experience. For instance, many physicians favored cephalosporins over penicillin to avoid skin testing requirements or selected macrolides based on the mistaken belief that they carry a lower risk of resistance, despite resistance patterns in China suggesting otherwise. These findings underscore the importance of clinical decision support systems, continuous professional development, and stewardship oversight to bridge the gap between knowledge and clinical practice.

Comparable challenges were observed among veterinary students in Ethiopia (Nyokabi et al.). While targeted educational programs improved understanding of antimicrobial stewardship, responsible antimicrobial use, and the interconnectedness of human, animal, and environmental health, important knowledge gaps regarding One Health, rational antimicrobial prescribing, and AMR remained. In addition, students identified effective communication with farmers and the public as a persistent challenge. This observation emphasizes that effective stewardship requires not only technical knowledge, but also strong communication, leadership, and community engagement skills. Pereira et al. further highlight the need to extend One Health stewardship efforts beyond healthcare professionals, as antimicrobial use among fighting-cock owners in Timor-Leste was common despite limited awareness of AMR.

One Health stewardship should move beyond guideline dissemination and encompass diverse stakeholder groups. It must include professional mentorship and behavioral interventions to ensure that evidence-based practices are implemented in clinics and on farms. Furthermore, audit and feedback mechanisms should be integrated to strengthen stewardship oversight.

The future of infection prevention will depend on innovative and sustainable interventions that complement conventional antimicrobial strategies. In this Research Topic, the systematic review by Sodre et al. highlights the potential of *Limosilactobacillus fermentum* cell-free supernatants (CFS) as a promising One Health-aligned approach. CFS’s broad-spectrum antimicrobial, antibiofilm, and immunomodulatory properties suggest potential applications across human, animal, and environmental sectors. The ability of CFS to inhibit biofilm formation is particularly relevant as biofilms contribute to the persistence and spread of MDR pathogens in both clinical and environmental settings. Moreover, CFS may help maintain intestinal barrier integrity, modulate the gut microbiota, and strengthen host defenses. However, more work is needed to standardize methodologies and validate *in vivo* efficacy and clinical effectiveness before implementation.

Beyond biological innovations, effective AMR mitigation also depends on strategic communication and public engagement. Parveen et al. analyzed AMR-related Instagram content and showed that although social media can reach large audiences, especially younger populations, it is still underutilized in AMR awareness. These findings highlight an important opportunity to strengthen public engagement by targeting audiences that increasingly rely on social media for health information. Educational and informative messages generate high engagement; however, incorporating personal stories or humor may further enhance audience engagement and broaden message reach. Digital communication plays an important role in AMR stewardship efforts. Digital platforms should be employed to transform AMR awareness from a purely academic topic into an urgent and relatable public health issue.

The studies in this Research Topic emphasize the importance of coordinated AMR communication across disciplines and sectors to facilitate the translation of scientific evidence into practice. Strengthening communication strategies across One Health stakeholders is essential to promote responsible antimicrobial use and foster collaboration across human, animal, and environmental health.

Future efforts should focus on education, implementation of clinical decision support systems, behavioral interventions that support antimicrobial stewardship, and the development of innovative antimicrobial approaches to achieve sustainable AMR mitigation within the One Health framework. Infection prevention should not be viewed solely as a medical problem but should also incorporate educational, behavioral, digital, and innovative tools to support sustainable One Health solutions.

